# Radiation-Induced Esophagitis is Mitigated by Soy Isoflavones

**DOI:** 10.3389/fonc.2015.00238

**Published:** 2015-10-21

**Authors:** Matthew D. Fountain, Lisa M. Abernathy, Fulvio Lonardo, Shoshana E. Rothstein, Michael M. Dominello, Christopher K. Yunker, Wei Chen, Shirish Gadgeel, Michael C. Joiner, Gilda G. Hillman

**Affiliations:** ^1^Department of Immunology and Microbiology, Karmanos Cancer Institute, Wayne State University School of Medicine, Detroit, MI, USA; ^2^Department of Oncology, Karmanos Cancer Institute, Wayne State University School of Medicine, Detroit, MI, USA; ^3^Department of Pathology, Karmanos Cancer Institute, Wayne State University School of Medicine, Detroit, MI, USA

**Keywords:** radiation, soy isoflavones, esophagitis, histopathology, radioprotection

## Abstract

**Introduction:**

Lung cancer patients receiving radiotherapy present with acute esophagitis and chronic fibrosis, as a result of radiation injury to esophageal tissues. We have shown that soy isoflavones alleviate pneumonitis and fibrosis caused by radiation toxicity to normal lung. The effect of soy isoflavones on esophagitis histopathological changes induced by radiation was investigated.

**Methods:**

C57BL/6 mice were treated with 10 Gy or 25 Gy single thoracic irradiation and soy isoflavones for up to 16 weeks. Damage to esophageal tissues was assessed by hematoxylin–eosin, Masson’s Trichrome and Ki-67 staining at 1, 4, 10, and 16 weeks after radiation. The effects on smooth muscle cells and leukocyte infiltration were determined by immunohistochemistry using anti-αSMA and anti-CD45, respectively.

**Results:**

Radiation caused thickening of esophageal tissue layers that was significantly reduced by soy isoflavones. Major radiation alterations included hypertrophy of basal cells in mucosal epithelium and damage to smooth muscle cells in muscularis mucosae as well as disruption of collagen fibers in lamina propria connective tissue with leukocyte infiltration. These effects were observed as early as 1 week after radiation and were more pronounced with a higher dose of 25 Gy. Soy isoflavones limited the extent of tissue damage induced by radiation both at 10 and 25 Gy.

**Conclusion:**

Soy isoflavones have a radioprotective effect on the esophagus, mitigating the early and late effects of radiation injury in several esophagus tissue layers. Soy could be administered with radiotherapy to decrease the incidence and severity of esophagitis in lung cancer patients receiving thoracic radiation therapy.

## Introduction

Radiation-induced toxicity to normal esophageal tissue is a dose-limiting side effect of radiotherapy for lung cancer and head and neck cancer (1–5). Radiation-induced toxicity to the esophagus can also be exacerbated by the addition of one or more chemotherapeutic drugs to the radiation treatment (1, 3–5). Concurrent chemoradiation therapy, consisting of fractionated radiation doses of 1.8–2 Gy per day delivered over a period of 6–7 weeks with concurrent chemotherapy, is the standard of care in patients presenting with locally advanced non-small cell lung cancer (NSCLC) (6, 7). Several trials have shown that concurrent chemotherapy with radiotherapy is superior to sequential chemotherapy followed by radiotherapy, achieving an absolute improvement in survival of 10% at 2 years (6, 7). Although, concurrent chemoradiation therapy results in improved survival outcomes, it is associated with increased toxicity, specifically acute esophagitis (3, 4, 8).

Esophagitis is an early side effect of radiation or chemoradiation, causing pain, difficulties in swallowing with potential malnutrition and dehydration. Strictures may develop in the chronic phase as a result of radiation-induced fibrosis (9). Esophagitis presents with odynophagia (irritation and burning sensation upon swallowing) and dysphagia (difficulties in the function of swallowing). Odynophagia symptoms usually have a rapid onset, and can be dose-limiting, presenting a serious challenge for radiation oncologists and can result in “premature” (early) termination of radiation treatment or breaks in therapy (3, 10–12). Odynophagia can be attributed to histological changes from radiation injury, including rapid mucosal epithelium (EPI) depletion, limited basal cell proliferation for epithelial repopulation, tissue inflammation, and tissue ulceration (12–14). Alternatively, dysphagia results from an acute or chronic tissue response to radiation injury and produces a mechanical restriction (and subsequent discomfort) in the action of swallowing (11, 12). Radiation Therapy Oncology Group (RTOG) and NCI CTCAE version 4 criteria scored acute esophagitis with toxicity grades as Grade 1 defined by asymptomatic or mild dysphagia or odynophagia (may require topical anesthetic or non-narcotic analgesic, soft diet); Grade 2 with symptomatic esophagitis, altered eating/swallowing (may require narcotic analgesics, puree or liquid diet); Grade 3 with severely altered eating/swallowing with dehydration or weight loss (N-G feeding tube, TPN, i.v. fluids or hospitalization indicated); Grade 4 with life-threatening consequences, with complete obstruction, ulceration, perforation, fistula, and urgent operative intervention if necessary (3, 10).

Analysis of RTOG trials reported that 95% of patients receiving concurrent chemoradiation for NSCLC developed some level of radiation esophagitis (grades 1–4) and 33% of patients experienced severe esophagitis (Grade ≥3) peaking within the first or second month of radiotherapy (3). Recent international meta-analysis for patients receiving concurrent chemoradiation reported about fivefold increase in the risk of acute radiation esophagitis and symptomatic radiation pneumonitis in 15–40% of patients compared with patients receiving sequential treatment (4, 5). These symptoms could lead to early interruption of radiation treatment, limiting the efficacy of radiation therapy. Therefore, it is imperative to design strategies to alleviate the clinical symptoms of radiation-induced toxicity to the normal esophagus.

In the current study, we have investigated a complementary approach to radiotherapy using soy isoflavones to reduce esophagitis. We hypothesized that soy isoflavones could provide radioprotection to normal esophageal tissues from radiation-induced tissue damage, based on our previous studies showing mitigation of radiation-induced pneumonitis and fibrosis by soy isoflavones. Soy isoflavones consist of a mixture of genistein, daidzein, and glycitein plant estrogens extracted from soy beans and are non-toxic dietary compounds that have demonstrated chemoprevention effects and anti-cancer properties (15). We have demonstrated that soy isoflavones enhance radiation-induced cancer cell killing *in vitro* and *in vivo* in tumor models of prostate cancer, renal cancer, and lung cancer (16–19). In addition to radiosensitizing tumor cells, we have recently shown that soy isoflavones could also act as radioprotectants of normal lung tissues when given in conjunction with radiation therapy (19, 20). In pre-clinical studies, soy isoflavones reduced the extent of pneumonitis and fibrosis caused by radiation damage to normal lung tissue (18–20). This differential effect of soy isoflavones on cancer cells versus normal tissue was shown in an orthotopic murine model of lung cancer, demonstrating that soy increased radiation-induced destruction of lung tumor nodules while simultaneously mitigating vascular damage, inflammation, and fibrosis caused by radiation in normal lung tissue (18–20). Further studies conducted in naïve mice receiving a high dose of 12 Gy thoracic irradiation in conjunction with soy isoflavones given pre- and post-radiation, confirmed their radioprotective effects (20). Soy isoflavones mitigated radiation injury to skin and hair follicles, protected respiratory function, decreased pneumonitis by limiting the thickening of alveolar septa and inflammatory infiltration, and reduced the extent of lung fibrosis caused by radiation at late time points (20). These findings within the normal lung parenchyma are indicative of early and late effects of radiation-induced tissue injury and subsequent inflammatory response, and support a radioprotective role for soy isoflavones. As suggested by the lung studies, soy isoflavones have the capacity to affect multiple aspects of the inflammatory response to radiation injury, and could potentially play role in alleviating tissue injury in other thoracic organs.

Given the potential severity of esophagitis caused by radiation in lung cancer patients, we have now investigated whether soy isoflavones could reduce the tissue damage in normal esophagus. These studies were conducted in naïve immunocompetent mice, using conditions of a single high dose of thoracic radiation at 10 Gy or 25 Gy to detect significant tissue damage. To characterize the histopathological changes of radiation-induced damage in esophagus and investigate the effect of soy isoflavones on esophagitis, we have examined esophageal tissues using histological and immunohistochemistry methods. Histopathological changes induced by radiation were observed in multiple tissue layers of the esophagus, including disruptions in the mucosal epithelium, lamina propria, muscularis mucosa and submucosa tissue layers. Our studies showed that soy isoflavones reduced the extent of damage to the various esophageal tissue layers and confirmed a role for soy isoflavones as a complementary and safe approach to improve the efficacy of radiotherapy for lung cancer.

## Materials and Methods

### Mice

Female C57BL/6 mice of 5–6 weeks old (Harlan, Indianapolis, IN, USA) were housed and handled in animal facilities accredited by the Association for Assessment and Accreditation of Laboratory Animal Care (AAALAC). Animal protocol was approved by the Institutional Animal Care and Use Committee (IACUC).

### Soy Isoflavones

The soy isoflavones mixture G-4660 used is a pure extract of 98.16% isoflavones from soybeans consisting of 83.3% genistein, 14.6% daidzein, and 0.26% glycitein (manufactured by Organic Technologies and obtained from NIH). The soy isoflavone mixture was dissolved in dimethyl sulfoxide (DMSO) and diluted 1:20 in sesame seed oil just prior to gavage to avoid irritation of the esophagus by DMSO. Mice were orally treated with soy isoflavones by gavage. Mice in the control and radiation-alone groups received vehicle alone. No toxicity was observed in mice treated with soy isoflavones prepared in DMSO and sesame seed oil with treatment given 5 days a week by gavage for up to 16 weeks in multiple experiments.

### Thoracic Irradiation

Radiation was delivered at a single dose of 10 Gy or 25 Gy to the mouse thorax, including esophagus, bilateral lungs, and mediastinum. Three anesthetized mice, in jigs, were positioned under a 6.4 mm lead shield with three cut-outs in an aluminum frame mounted on the X-ray machine to permit selective irradiation of the lung in three mice at a time, as previously described (18–20). Radiation dose to the thorax and scattered dose to areas of the mouse outside of the radiation field were carefully monitored. Dose rate was 101 cGy/min and HVL was 2 mm Cu. Photon irradiation was administered using a Siemens Stabilipan X-ray set (Siemens Medical Systems, Inc.) operating at 250 kV, 15 mA, with 1 mm copper beam filtration at a distance of 47.5 cm from the target.

### Experimental Protocol

Mice were pre-treated with oral soy isoflavones each day for 3 days at a dose of 5 mg/day (equivalent to 250 mg/kg). On day 4, the thorax was selectively irradiated with a dose of 10 Gy or 25 Gy. Soy treatment was continued on a daily basis for 5 more days at 5 mg/day. Then, mice were treated with a lower soy isoflavone dose of 1 mg/day (equivalent to 50 mg/kg), given daily 5 days a week for up to 16 weeks. The rationale for giving a higher dose of soy isoflavones for pre-treatment and just after radiation is to optimize the effect of soy based on our previous studies (18–20).

### Esophageal Tissue Preparation for Histology

Mice were sacrificed at different time points between 1 and 16 weeks after irradiation. Esophagi were resected, fixed in 10% formalin, and curved into a U shape for embedding in a vertical position in paraffin and transverse tissue sections were cut. Esophagus sections were stained with hematoxylin–eosin (H&E) and examined using a Nikon E800 microscope. Morphometric measurements of inner esophageal tissue layers were performed using Image-ProPlus version 6.2 software. The effect of radiation and soy on esophageal layers was evaluated using Masson trichrome (MT) stain for detecting collagen fibers. Histological findings were quantified using a subjective 4-tiered scale from weak (±), moderate (+), strong (++), and heavy (+++). Four mice per group were used for data analysis in two series of repeated experiments.

### Immunohistochemistry

Sections of esophagi were stained by immunohistochemistry. Sections were blocked with IHC Tek Antibody Diluent, and then incubated with primary purified monoclonal antibodies directed against α smooth muscle actin (α-SMA) (1:4000), CD45 (1:200), and Ki-67 (1:350) followed by biotinylated secondary antibodies (1:300). Staining was amplified with the avidin-biotin system immunoperoxidase technique. Esophageal layers were examined on a Nikon E800 microscope. Four mice per group were used for data analysis in two series of repeated experiments.

### Statistical Analysis

Differences in the thickness of inner esophageal tissue layers among the various treatment groups were analyzed in four mice per group at weeks 4, 10, and 16. To compare the treatment effect at each time point, a linear mixed-effects model allowing for nested random effects was used (21). A *p*-value of less than 0.05 was considered significant. The statistical software R (22) and package nlme (23) was used.

## Results

### Soy Isoflavones Reduce Radiation Damage to Esophageal Layers

**Figure 1:**
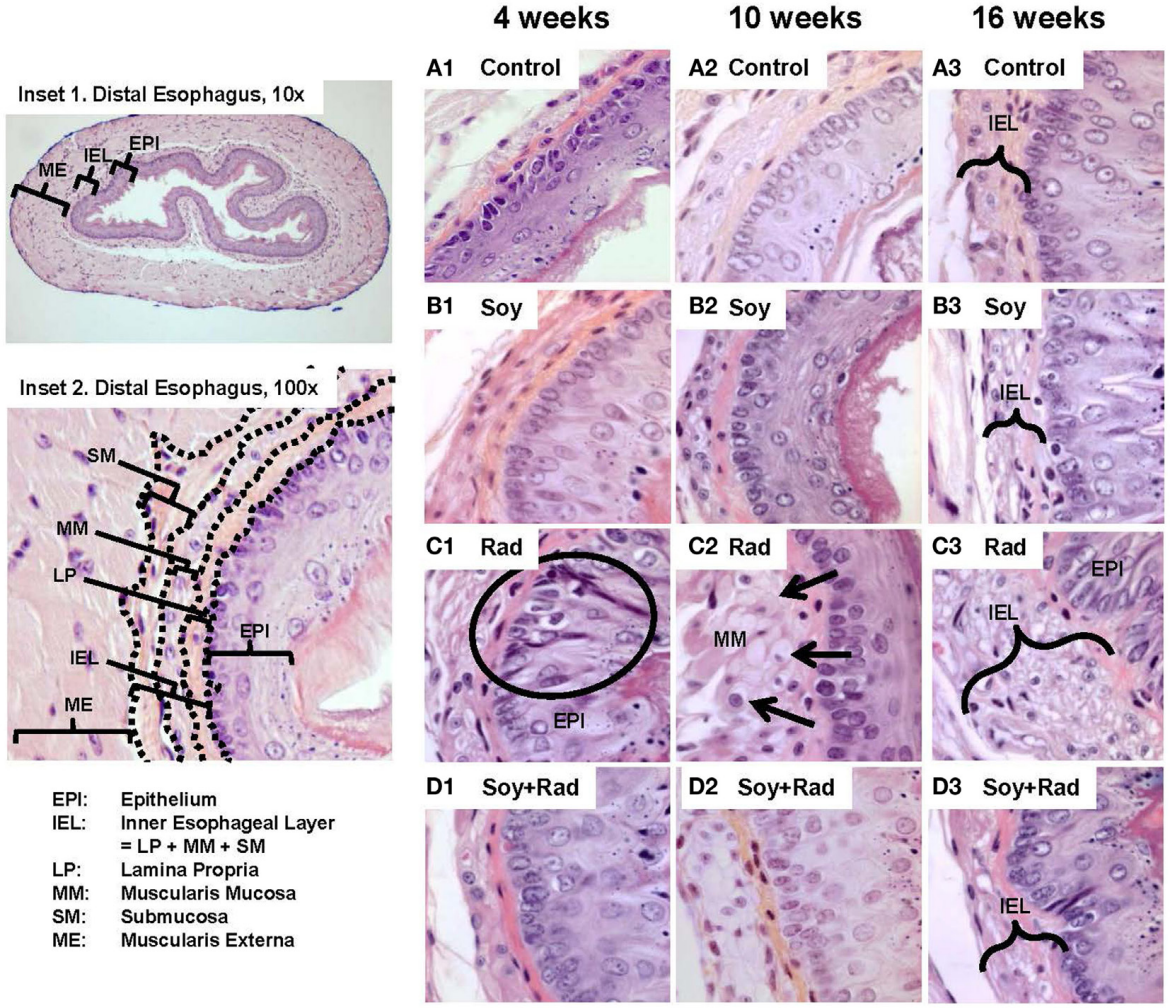
**H&E staining of esophagus tissue sections treated with soy, radiation, and soy **+** radiation**. Esophageal tissue sections were obtained from control mice and mice treated with soy or radiation (Rad) or soy + radiation (Soy + Rad) at 4, 10, and 16 weeks after radiation. Sections were stained with H&E. Inset 1 shows a transverse section of normal esophagus at low magnification (10×). Inset 2 shows esophageal tissue layers starting from the luminal interface, including the mucosal epithelium (EPI), lamina propria (LP), muscularis mucosae (MMs), submucosa (SM), and the muscularis externa (ME) (×100). Because lasting alterations induced by radiation were observed in LP, MM, and SM, these three layers were grouped as inner esophageal layer (IEL). **(A)** Sections from control mice showing normal morphology of the epithelium and IEL. **(B)** Sections from mice treated with soy are showing mild changes in IEL and EPI. **(C)** Esophagi from mice treated with radiation, at 4 weeks, showing alterations in the EPI with disruptions in the alignment of epithelial cells and basal cell hyperplasia (circle in 1). In the MM layer, consisting of smooth muscle cells, hypertrophy and cytoplasmic vacuolization were observed (arrows in 2). Overall increase in the thickening of IEL and extensive vacuolization were observed at 4, 10, and 16 weeks after radiation (brackets in 3). **(D)** Sections from mice treated with soy + radiation showing attenuation in these findings, mostly in LP and MM layers. All magnifications ×100.

To assess the effects of irradiation and soy isoflavones on normal esophagus, the esophagus was resected at 4, 10, and 16 weeks after treatment with either soy alone, radiation alone, or both radiation and soy isoflavones. Esophageal tissues were processed for histological examination. Representative images are presented in Figure 1. A transverse section of the esophagus shows tissue layers lining the lumen and extending into the muscle layers of the muscularis externa (ME, Figure 1, Inset 1). Esophageal tissue layers include the mucosae consisting of stratified squamous epithelium (EPI), the lamina propria (LP), which is a loose connective tissue, the muscularis mucosae (MM) composed of longitudinally organized bundles of smooth muscle fibers, and the submucosa (SM) consisting of a thin layer of connective tissue lining the muscularis externa (ME) (Figure 1, Inset 2). Because radiation caused thickening of LP, MM, and SM, these three layers were grouped and defined as inner esophageal layer (IEL) (Figure 1, Inset 2). To evaluate the differences in IEL between treatment groups, morphometric measurements of IEL were performed (Table 1A). At all time points, esophagus from control mice showed normal esophageal tissue layer structure and integrity, including a healthy mucosal EPI, a thin, dense and continuous LP, and small smooth muscle cells within the MM (Figure 1A1,2,3) although a gradual increase in IEL measurements was observed with time by 4 months (Table 1A). Treatment with soy isoflavones did not show alterations in either mucosal EPI or IEL, which were comparable to control esophagi (Figure 1B1,2,3; Table 1A). By contrast, radiation-treated esophagi showed marked alterations in tissue layers that were observed at 4, 10, and 16 weeks after radiation. Mucosal EPI showed disruption in the basal cell layer alignment, including hyperplasia and hypertrophy of the basal cells (see circle in Figure 1C1). Radiation caused progressive enlargement of MM, secondary to hypertrophy and vacuolization of smooth muscle cells (arrows in Figure 1C2). The IEL was much thicker as seen already at 4 weeks, and further increased at 10- and 16-week time points (bracket in Figure 1C3). Morphometric measurements confirmed a significant increase in IEL thickness compared to control at all time points (*p* < 0.001, Table 1A). Esophagi from mice treated with soy isoflavones before and after radiation showed minimal tissue damage with a mucosal EPI and IEL comparable to that in normal esophagus (Figure 1D1–3), and no significant changes in IEL measurements were observed compared to controls (*p* > 0.1, Table 1A). Soy isoflavones reduced the extent of radiation-induced IEL thickening (Figure 1D1–3). Measurements of IEL thickness showed that at 10 weeks the IEL was significantly smaller in soy + radiation compared to radiation (*p* < 0.001, Table 1A) and was comparable to control IEL (*p* > 0.05, Table 1A). At time points of 4 and 16 weeks, the IEL was marginally yet significantly reduced in radiation + soy compared to radiation (*p* = 0.052–0.053, Table 1A).

**Table 1:** **Assessment of histological alterations in esophageal tissues**.

	Treatment	4 weeks	10 weeks	16 weeks
A. IEL thickness (mean μm ± SEM)	Control	15.77 ± 0.40	22.74 ± 0.58	25.13 ± 0.54
	Soy	20.58 ± 0.59	21.80 ± 0.64	30.24 ± 1.08
	Radiation	30.66 ± 0.67	41.54 ± 1.18	40.43 ± 0.91
	Soy + Rad	25.54 ± 0.67	27.03 ± 0.74	33.28 ± 0.80
B. Collagen density in LP	Control	++	++	++
	Soy	++	++	++
	Radiation	+	+	+
	Soy + Rad	++	++	++
C. Smooth muscle cell hypertrophy in MM	Control	±	±	±
	Soy	±	±	+
	Radiation	++	+++	+++
	Soy + Rad	±	+	++

**Figure 2:**
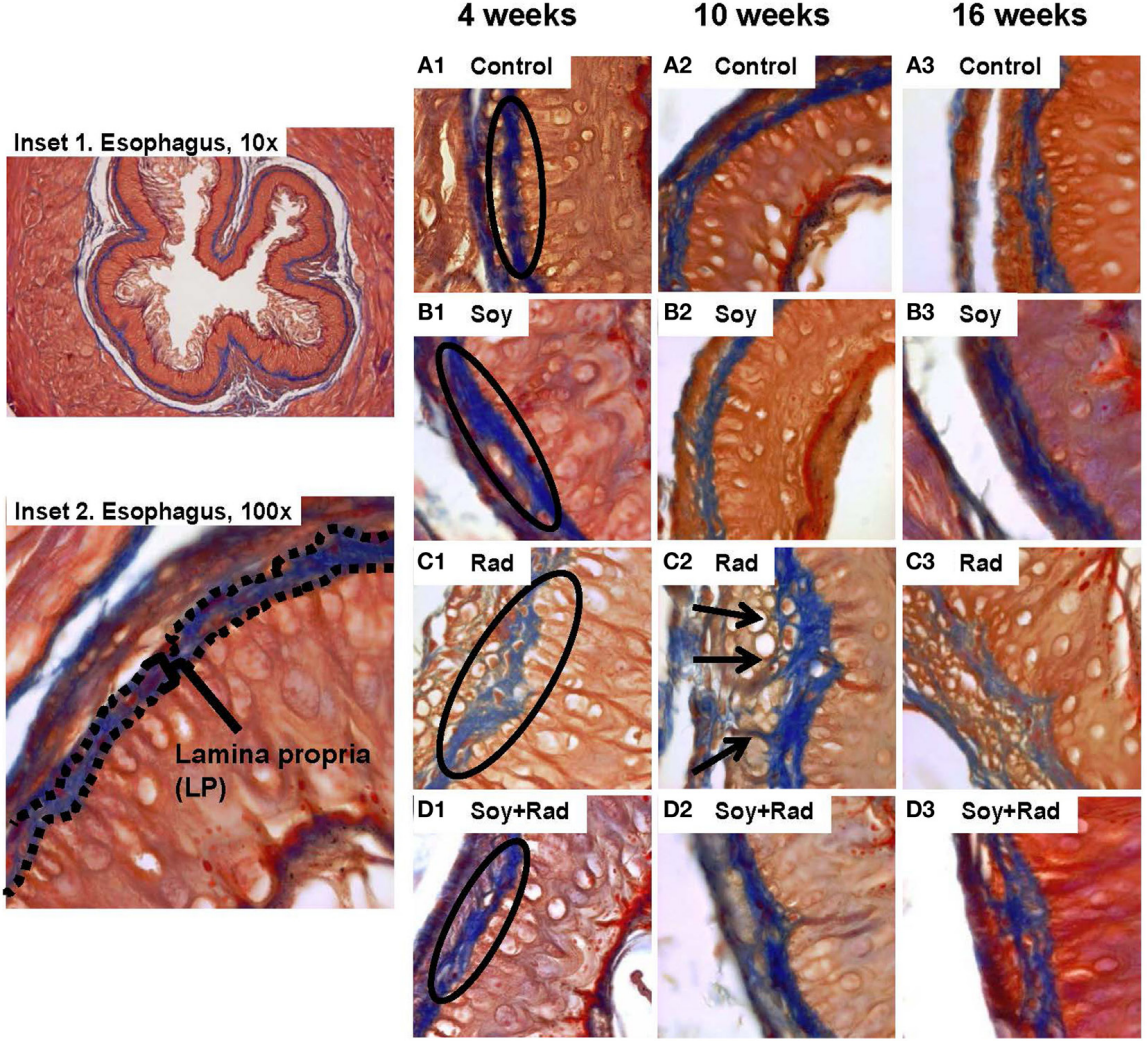
**Effect of soy and radiation on connective tissue layers of esophagus**. Esophageal sections obtained from the experiments described in Figure 1 were stained with Masson’s Trichrome for detection of collagen fibers within the LP and SM connective tissues. Insets 1 and 2 show normal esophagi with collagen staining in LP and SM below the negative staining of MM. **(A)** Sections from control mice showed similar collagen fibers distribution limited to LP and SM as labeled in A1. **(B)** Sections from mice treated with soy showed staining patterns comparable to controls. **(C)** Sections from mice treated with radiation showed disruptions in the LP (labeled in C1) and infiltration of collagen fibers focally into the MM with entrapment of hypertrophic smooth muscle cells (arrows in C2). **(D)** Sections from mice treated with soy + radiation showed reduced collagen infiltration into MM smooth muscle compared to radiation and a higher density of fibers (labeled in D1) that is comparable to LP of control and soy-treated esophagus. All magnifications ×100.

**Figure 3:**
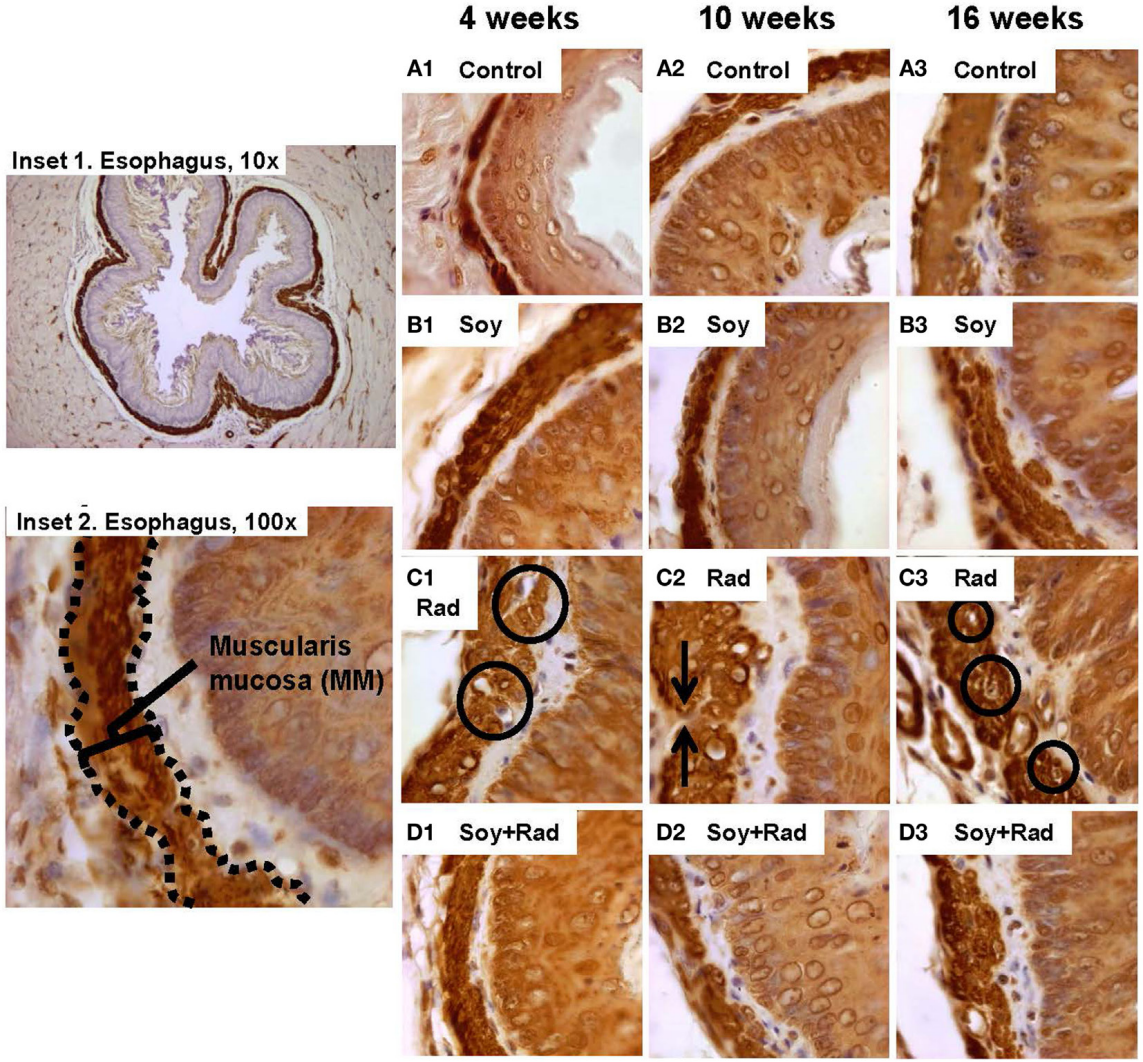
**Effect of soy and radiation on smooth muscle cells in muscularis mucosae (MMs)**. Esophagus tissue sections obtained from the experiments described in Figure 1 were stained with anti-αSMA by IHC for detection of smooth muscle cells in MM. Insets 1 and 2 show a low and high power view of normal esophagus highlighting MM. **(A)** Sections from control mice and **(B)** sections from mice treated with soy showed comparable morphology and thickening of MM. **(C)** αSMA staining confirmed thickening of LP and MM caused by radiation and smooth muscle cell enlargement (circles in C.1), large empty vacuoles, and condensation of nuclei in MM (circles in C3). Interruptions in the integrity of MM layer were also observed (arrows in C.2). **(D)** Sections from mice treated with soy + radiation showed reduced smooth muscle cell hypertrophy and limited disintegration of MM compared to radiation alone. All magnifications ×100.

*(A). Using Image Pro Plus software analysis of H&E slides, morphometric measurements of inner esophageal layer (IEL) thickness were performed at 100× magnification for each mouse esophagus at 4, 10, and 16 weeks after radiation. Data were computed from four representative mice in two separate experiments. Five measurements were taken in five random fields per mouse, yielding 25 measurements per mouse and 100 measurements for four mice per treatment group, and the means ± SEM are reported. (B,C). Histological findings from Masson’s trichrome staining for collagen density and smooth muscle cells presented in Figures 2 and 3 were scored. The extent of collagen density within the lamina propria (LP) and smooth muscle cell hypertrophy of the muscularis mucosae (MMs) were scaled from weak (±), moderate (+), strong (++), to heavy (+++)*.

### Soy Isoflavones Reduce Disruptions of Lamina Propria Connective Tissues Caused by Radiation

Previous studies in our lab showed that soy isoflavones decrease the pulmonary fibrosis induced by radiation (18–20). In order to evaluate the effect of radiation and/or soy isoflavones on collagen fibers within the LP and SM, tissue sections were stained with Masson’s Trichrome. Esophagi from control mice showed dense blue, collagen in LP at all time points with few thin wisps in SM (circles in Figure 2A1–3). Similar findings were seen in mice treated with soy isoflavones alone (circles Figure 2B1–3). Irradiated esophagi revealed that the density of collagen fibers was reduced in the LP at 4 weeks (circle in Figure 2C1) and collagen fibers were seen infiltrating into MM layer at 10 and 16 weeks (arrows in Figure 2C2,3). Esophagi from mice treated with irradiation and soy isoflavones showed increased collagen density in LP, and reduced infiltration of collagen in the MM. These findings were comparable to that of control and soy isoflavone treated mice, predominately at 4 and 10 weeks after radiation (Figure 2D1–3). Assessment of alterations in LP collagen density is scored and provided in Table 1B.

### Soy Isoflavones Decrease Smooth Muscle Cell Destruction Induced by Radiation

H&E staining and Masson’s Trichrome stain of esophagus tissues showed hypertrophy and vacuolization of smooth muscle cells in MM. In order to evaluate smooth muscle cell hypertrophy within the MM tissue layer, IHC staining with αSMA was performed. Comparable MM thickness in control and soy-treated mice was seen with a slight increase in MM thickness over time (Figures 3A,B1–3). Following ­radiation, IHC confirmed H&E findings with hypertrophied large smooth muscle cells showing cytoplasmic vacuolization at all time points (circles in Figure 3C1,3). Additionally, irradiated esophagi showed discontinuity within the MM layer (arrows in Figure 3C2). Esophagi from mice treated with combined radiation and soy isoflavones showed reduced smooth muscle cell hypertrophy, with cell size comparable to control, and limited fragmentation of MM smooth muscle throughout all time points after radiation treatment (Figure 3D1,2,3). Assessment of alterations in MM smooth muscle cell hypertrophy is scored in Table 1C.

### Soy Isoflavones Reduce Leukocytic Infiltration Within Esophageal Tissue Layers after Radiation

**Figure 4:**
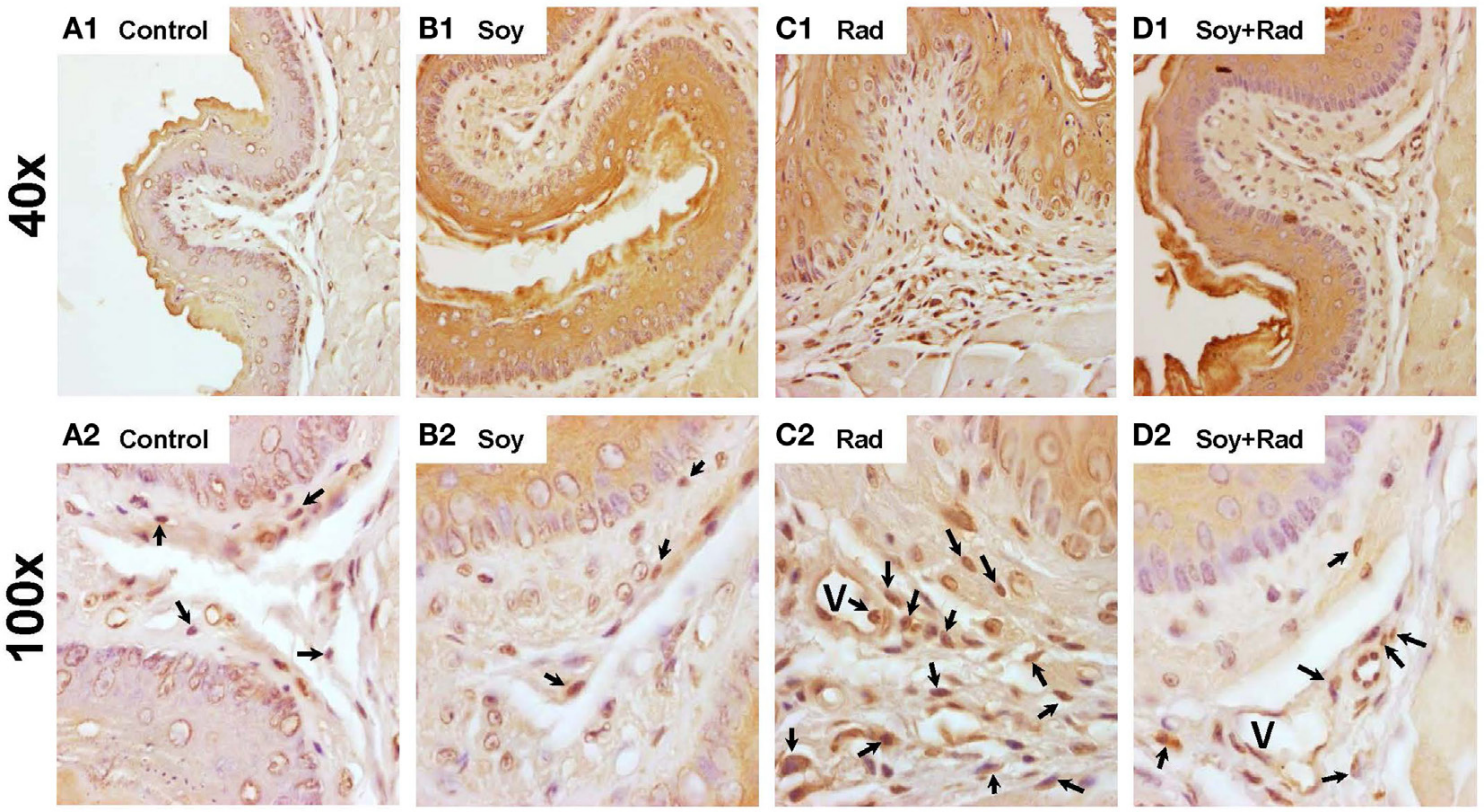
**Leukocyte infiltration in esophageal tissues treated with soy and radiation**. Esophagi tissue sections were obtained from control mice and mice treated with soy or radiation (Rad) or soy + radiation (Soy + Rad) at 4 weeks after radiation. Sections were stained with anti-CD45 by IHC for detection of leukocytes in IEL. [**(A)** 1,2] Sections from control mice showed scattered CD45+ cells within the IEL layers. [**(B)** 1,2)] Similar distribution of CD45+ leukocytes in mice treated with soy compared to control. [**(C)** 1,2)] Sections from mice treated with radiation showing a prominent infiltration of CD45+ leukocyte infiltration in all layers of IEL (arrows in C2). [**(D)** 1,2] Sections from mice treated with soy + radiation with much lower levels of CD45+ cell infiltrates comparable to those of control and soy-treated esophagus. (magnifications ×40 for A1, B1, C1, D1; and ×100 for A2, B2, C2, D2).

To evaluate inflammatory infiltration induced by radiation and the effect of soy isoflavones, esophageal tissue sections, obtained at 4 weeks, were stained by IHC using the pan-leukocyte CD45 marker. Esophagi from untreated mice and mice treated with soy isoflavones showed a normal distribution of CD45 + leukocytes scattered within the IEL layers (Figures 4A,B). Irradiated esophagus tissues showed a major infiltration of CD45 + leukocytes in all layers of IEL often organized in focal clusters (arrows Figure 4C2). Esophagi treated with radiation and soy isoflavones showed much lower levels of CD45+ cell infiltrates comparable to those of control and soy-treated esophagus (Figure 4D1,2). These data were reproducible and observed in four mice per treatment group.

### Soy Isoflavones Mitigate Early Effects of Radiation-Induced Damage on the Mucosal Epithelium

**Figure 5:**
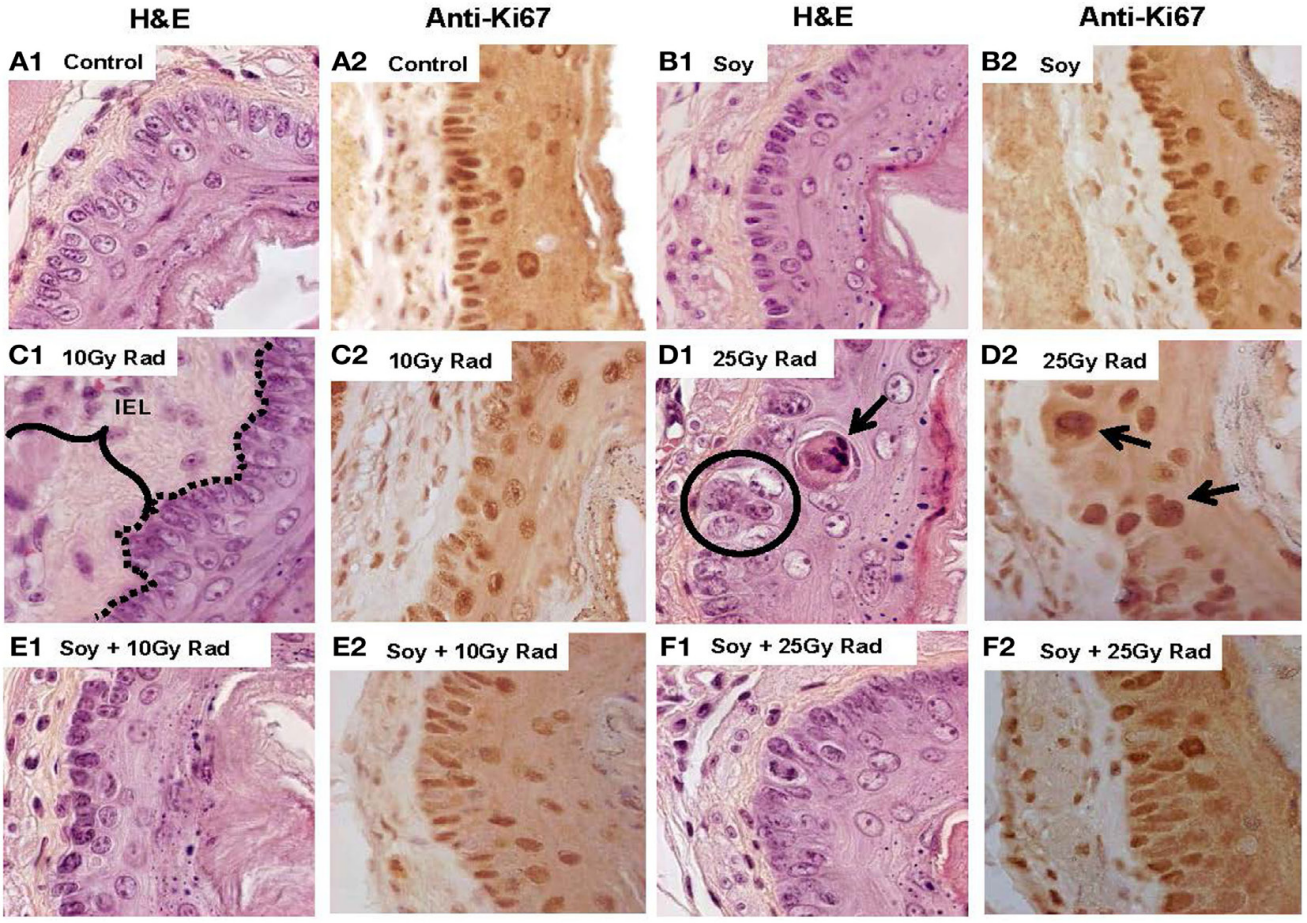
**Early effects of soy on esophageal tissues treated with 25 Gy radiation compared to 10 Gy radiation**. Esophagi were obtained from control mice and mice treated with soy or radiation (Rad) or soy + radiation (Soy + Rad) at 1 week after a radiation dose of 10 or 25 Gy. Sections were stained with H&E (series 1) and Ki-67 (series 2). Sections from control mice **(A)** and sections from mice treated with soy **(B)** both showed normal morphology and active proliferation of mucosal epithelial cells. **(C)** Sections from mice treated with 10 Gy radiation showed enlargement of IEL and disruptions in basal cell basement membrane continuity (dotted lines in C.1) and decreased proliferation (C.2). **(D)** A higher dose of 25 Gy radiation caused greater alterations in the EPI mucosal layer, including basal cell hyperplasia, enlargement of epithelial cells (circles in D.1), and apoptotic keratinocytes in the parabasal layer (arrows in D.1). Ki-67+ cells showed staining of large nuclei but an overall decrease in the number of dividing cells (D.2). **(E,F)** Sections from mice receiving combined treatment of 10 or 25 Gy radiation with soy showed decreased alterations in mucosal basal cell morphology and size, improved basal cell division, and decreased thickening of the IEL.

We have described the histopathological changes occurring in response to radiation, and mitigation by soy isoflavones at 4 to 16 weeks after radiation treatment and at a single dose of 10 Gy radiation. The effects of soy isoflavones at an early time point after radiation and at a higher dose of radiation were tested to determine whether alterations caused by radiation can be detected earlier and are exacerbated by a higher radiation dose. Esophageal tissue sections, stained with H&E or Ki-67, were evaluated at 1 week after radiation from mice receiving either 10 Gy or 25 Gy irradiation alone or in combination with soy isoflavones. Untreated esophagi showed normal tissue layers at 1 week after radiation (Figure 5A1) and active proliferation of mucosal epithelial cells (5A2). Esophagi from mice treated with soy isoflavones showed normal, undisturbed tissue layers with dividing epithelial cells, similar to control (Figure 5B1,2). As previously shown at later time points (Figure 1C), increased thickening of the IEL (bracket in 5C2) and disruptions in the continuity of mucosal basal cells were already observed at 1 week after 10 and 25 Gy radiation. The number of dividing epithelial cells was markedly reduced by 10 Gy (Figure 5C2) while 25 Gy not only showed a paucity of normal size dividing epithelial cells but showed major alterations with apoptotic keratinocytes (Figure 5B1), enlarged and hyperplastic cells with large dividing nuclei probably due to formation of degenerating giant cells, characteristic of radiation effects (Figure 5D1,2). By contrast, esophagi from mice treated with radiation and soy isoflavones showed limited alterations in basal cell morphology and size with no disruption in the alignment along the basement membrane, and reduced thickening of IEL (Figures 5E,F,1). Soy isoflavones improved epithelial cell division (Figure 5E2); however, at 25 Gy, some areas still showed some enlarged, dividing cells (Figure 5F2). These data were reproducible and observed in four mice per treatment group.

## Discussion

For NSCLC patients, radiation-induced esophagitis presents a major radiation dose-limiting challenge to radiation oncologists may result in dehydration, malnutrition, and even disruption of the treatment course (1–5). Nearly all patients experience some level of esophageal toxicity within the field of their radiation treatment, and present with varying severity of odynophagia and dysphagia as a result of tissue injury and inflammation, necessitating the need for intervention with effective therapeutics.

We have previously reported the radioprotective effect of soy isoflavones for lung tissue in lung tumor bearing mice and in naïve mice, mitigating radiation-induced pneumonitis and pulmonary fibrosis (18–20). To further investigate whether soy isoflavones could protect other thoracic organs in the field of radiation for NSCLC, in particular the esophagus, we have now explored the effect of radiation alone given at a single high dose of 10 Gy and combined with soy isoflavone supplementation on esophageal tissue layers. In an early response to radiation, the esophagus undergoes extensive tissue edema (12), which is a hallmark of radiation-induced tissue damage which has also been documented in other organs, including lung EPI and gut mucosa (24–26). Radiation produces a cyclic, chronic inflammatory response, which includes the deposition of collagen and reconstitution of extracellular matrix compartments (25, 27). Prolonged accumulation of collagen and extracellular matrix components explains long-term development of esophageal strictures, occurring months to years after radiation treatment (1, 2, 12, 27).

In the current pre-clinical study, major alterations in esophageal tissue layers caused by radiation injury were observed at both early and late time points up to 4 months after radiation. In particular, thickening of the IELs was observed from 4 to 16 weeks after radiation. The edematous response of esophageal tissues is well documented and esophageal swelling can be considered a contributor to dysphagia caused by radiation treatment, as suggested by endoscopy studies in patients (12). In our mouse studies, increased thickness of IEL layers following radiation was significantly reduced by complementing radiation with soy isoflavones, suggesting that soy isoflavones could limit the extent of tissue damage caused by radiation.

To analyze histopathological changes within the various tissues of the IEL, specific histological staining showed that radiation caused alterations in the esophageal connective tissue layers. Decreased density and altered distribution of collagen fibers were seen in the LP as well as infiltration of collagen fibers between hypertrophied smooth muscle cells in MMs. These alterations could be early events of the process of radiation-induced fibrosis. Fibrosis is a remodeling process to repair tissue damage by deposition of collagen and extracellular matrix into sites of damaged or necrotic tissue. (25, 27) Fibrotic accumulation of collagen over long periods of time – months to years – can lead to esophageal stricture formation and can cause dysphagia, a chronic symptom from radiation (1, 2, 12). Soy isoflavones showed a protective role on maintaining the density and integrity of collagen fibers in esophageal connective tissues and could play a role in decreasing the progression of long-term stricture formation as a result of radiation-induced fibrosis.

Specific staining of smooth muscle cells forming the MMs layer showed that these cells were sensitive to radiation resulting in hypertrophy, vacuolation, and cell death, leading to interruptions in the integrity of the MMs. This effect was seen at 4 weeks and persisted up to 16 weeks after radiation. However, smooth muscle cell disruption was reduced in tissues with combined treatment of radiation and soy isoflavones at all time points. Smooth muscle damage and loss in response to radiation have been observed in the walls of vessels and capillaries and are involved in vascular disruption (25). But specific studies investigating the pathological events related to smooth muscle cell destruction from radiation injury remain limited. Preservation of smooth muscle cell structure by soy isoflavones, both at the cellular level by reducing hypertrophy, and at the tissue level by reducing fragmentation, could provide functional conservation of MMs smooth muscle, limiting the development and progression of radiation-induced dysphagia in a clinical setting.

Inflammatory cell infiltration is known to be a normal response by various tissues to radiation injury in conjunction with tissue edema (25, 27, 28). In the esophagus, radiation caused a prominent infiltration of leukocytes in the IEL, but this process was limited when soy isoflavones were supplemented to radiation. Reduced inflammatory infiltrates were also documented in lungs treated with radiation and soy isoflavones in our previous studies (18–20). Further studies are ongoing to investigate the effect of soy isoflavones on mitigating the inflammatory response induced by radiation in normal tissues.

Finally, acute effects of radiotherapy on the esophagus involve sloughing of superficial layers of the squamous EPI in human studies (12). In mouse studies, we found that radiation injury to the mucosal EPI included disruptions in the basal cell layer alignment with hypertrophy of epithelial cells. Epithelial cell depletion, apoptotic keratinocytes, and abnormal giant cells with large nuclei were more pronounced with a higher single dose of 25 Gy irradiation, and were seen already at 1 week after radiation as confirmed by staining with Ki-67 proliferative marker. The regular arrangement of basal cells depend on intracellular tight junctions, which hold cells together and are the basis for maintaining mucosal tissue barrier functions (29). Soy isoflavones given in conjunction with radiation appeared to preserve the integrity of the basal cell layer of the mucosal EPI and fewer abnormal epithelial cells at both single doses of 10 and 25 Gy radiation at early and late time points. Soy isoflavones could therefore provide maintenance of mucosal barrier function and limit the depletion of the mucosal EPI, improving radiation-induced odynophagia. Indeed, in support of our findings, a review examining the role of flavonoids in intestinal tight junction regulation showed that genistein, the most bioactive isoflavone found in soy, protected intestinal tight junction barrier function by counteracting the disassembly of occludins induced by oxidative stress and acetylaldehyde (30). Other studies reported the role of genistein in reducing apoptosis induction in normal seminiferous tubule mucosal tissues (31) and intestinal mucosal tissues (32) in agreement with the effect of soy isoflavones in mitigating the effect of radiation damage on mucosal EPI in the esophagus. As strategies to alleviate esophagitis, phase II and III trials of amifostine, an organic thiophosphate and potent scavenger of oxygen free radicals, have shown mixed results in reducing esophagitis while causing nausea and vomiting in patients (9). By contrast, soy isoflavones have been shown to be safe natural compounds in all of our murine studies as well as in humans in controlled clinical trials (33).

Taken together, our study shows that soy isoflavones provide a radioprotective role by reducing radiation-induced damage to various tissue layers in the esophagus when radiation was delivered as a single high dose. Further studies are ongoing to compare the histopathology of single high dose to fractionated radiation and the effect of soy isoflavones. In a recent preliminary experiment, soy isoflavones also reduced alterations in esophageal tissues in mice receiving fractionated radiation and cisplatin which had greater damage in esophageal tissues by radiation and cisplatin compared to radiation alone. These findings suggest that soy isoflavones could mediate radioprotection in a murine model of concurrent chemoradiation, which is known to cause greater acute esophagitis in NSCLC patients. It is possible that the beneficial radioprotective effect of soy isoflavones at the histological level would translate also into a beneficial functional effect on esophageal motility and be integrated in clinical protocols to mitigate the harmful side effects of radiation. To this effect, we are currently conducting a Phase I clinical trial to evaluate the safety and efficacy of soy supplementation to conventional chemoradiotherapy for advanced stage III NSCLC disease.

## Author Contributions

MF: conducted all experiments and data analysis described throughout the manuscript. Major contributor to the writing of the manuscript and literature search. LA: contributed to experimental design, data analysis, and organization of the manuscript. FL: reviewed and analyzed all histology data, expert in pathology. SR: contributed to data analysis and imaging. MD: provided clinical expertise and participated in experimental design. CY: assisted with all experimental procedures in mice. WC: provided statistical expertise and analysis of data. SG: clinical oncologist, expert in lung cancer, esophagitis, provided clinical guidance. MJ: radiobiologist, collaborator on radioprotection studies and rationale for project. GH: principal investigator on grants and projects, designed, supervised the study, and major contributor to organization of the data and manuscript.

## Conflict of Interest Statement

The authors declare that the research was conducted in the absence of any commercial or financial relationships that could be construed as a potential conflict of interest.
